# Antibiotic Residues in Raw Cow Milk Collected from Smallholder Dairy Farms in Kasama and Mbala, Zambia

**DOI:** 10.3390/antibiotics14121197

**Published:** 2025-11-26

**Authors:** Goliath Eneya Zulu, Bernard Mudenda Hang’ombe, Geoffrey Mainda, Edgar Kayesa, Chitwambi Makungu, Abel Compbel Chipembo, Gilbert Nchima, Alberto Pondja, Niura Madalena Bila, Belisário Moiane

**Affiliations:** 1 Department of Animal Production and Food Safety, Faculty of Veterinary Sciences, Eduardo Mondlane University, Bairro Luis Cabral, Avenida de Moçambique, Km 1.5, Maputo P.O. Box 257, Mozambique; zulugoliathe@gmail.com (G.E.Z.); abelchipembo@uem.ac.mz (A.C.C.); apondja@uem.mz (A.P.); 2 Centre of Excellence in Agri-Food Systems and Nutrition (CE-AFSN), Eduardo Mondlane University, Praça 25 de Junho Edificio da Reitoria 5° andar, Maputo P.O. Box 257, Mozambique; 3 Department of Paraclinical Studies, School of Veterinary Medicine, The University of Zambia, Lusaka P.O. Box 32379, Zambia; bhangombe@unza.zm; 4 DVC Research & Innovation, Copperbelt University, Jambo Drive, Kitwe P.O. Box 21692, Zambia; 5 Department of Emergency Centre for Transboundary Animal Diseases, Food Agriculture Organization, Zenera Office Park, No. 2287/A, Lagos Rd and Lubuto Rd, Rhodes Park, Lusaka P.O. Box 30563, Zambia; Geoffrey.Mainda@fao.org (G.M.); Chitwambi.Makungu@fao.org (C.M.); 6 Department of Pathology, Central Veterinary Research Institute, Ministry of Fisheries and Livestock, Lusaka P.O. Box 33980, Zambia; Edgar.Kayesa@mfl.gov.zm; 7 Department of Biochemistry and Toxicology, Central Veterinary Research Institute, Ministry of Fisheries and Livestock, Lusaka P.O. Box 33980, Zambia; Gilbert.Nchima@mfl.gov.zm

**Keywords:** antimicrobial residues, antimicrobial resistance, Charm II receptor assay, dairy, one health, public health threat

## Abstract

**Background/Objectives:** The deposition of antibiotic residues in animal source foods has become a global public health threat. This study aimed to assess antibiotic class residues in raw cow milk from smallholder dairy farms in Mbala and Kasama, Zambia. **Methods:** A cross-sectional study was conducted, in which 93 milk samples (54 from Mbala and 39 from Kasama) were randomly collected from lactating cows on 56 farms between May and June, 2025. The samples were analyzed using the Charm II assay for beta-lactams, tetracyclines, macrolides, sulfonamides, and aminoglycosides. A total of 100 mL of milk was collected in sterile plain tubes, placed in a cooler box with ice packs, and transported to the district laboratory’s freezer and then delivered to the Central Veterinary Research Institute at (−18 to −20 °C), where they were stored at −20 °C. Statistical significance between districts was determined using Pearson’s chi-square, and associations between a district and the occurrence of antibiotic residues in milk were evaluated using logistic regression. Data were analyzed using Stata 14.2 at a 95% confidence level (*p* = 0.05). **Results:** A total of 91.4% (*n* = 85) of samples had antibiotic residues above EU/MRLs, with mean positive samples being 0.91 ± 0.28 and a significant association between a district and residue occurrence (OR = 0.086; *p* = 0.025). Approximately 44.1% of the samples had multiple antibiotic residues. Approximately 82.1% of samples from Kasama and 98.1% from Mbala had antibiotic residues (*p* = 0.006). Approximately 68.8% of samples had sulphonamides, and 58.1% macrolides, indicating their widespread use. Tetracyclines were 12.9%, beta-lactams 9.7%, and aminoglycosides 2.2%. **Conclusions:** A majority of milk samples had antibiotic residues above EU/MRLs, raising public health threats and necessitating the development and implementation of policies.

## 1. Introduction

The dairy sector is among the largest and fastest-growing segments of agriculture globally, with an engagement of about 150 million farm households focused on milk production, i.e., more than 750 million people, the majority of whom are in developing countries [1]. In 2023, 965.7 million tons of milk were produced globally, compared with 53.8 million tons in Africa [2]. In Zambia, smallholder dairy farming is practiced in a production system that integrates crop and dairy farming [3]. The dairy subsector in Zambia contributes a major part of the livestock sector. According to [3], in the Agriculture Consultative Forum report (ACF), Zambia had around 3000–4000 dairy producers in 2012, ranging from traditional to commercial producers, contributing approximately 253 million liters of milk to the national production capacity. Smallholder dairy producers in Zambia contribute about 50% of the marketed milk supply; large-scale commercial farmers supply 23%, while the remaining 27% is imported as milk and milk products [4]. In Zambia, raising livestock is one of the main agricultural pursuits. It contributes roughly 23% of the country’s total protein supply per person, with cattle providing 61% of this through milk and beef [5]. Dairy production is crucial to economic and sustainable development because it creates jobs, income, and food and nutritional security [6]. As reported in [7], revealed that Zambians consume 19–30 kg of milk annually per person, which is low for Sub-Saharan Africa (average of 30.2 kg) and far less than the WHO’s recommended 175 kg.

Antimicrobials have been used in livestock production for a number of purposes, including growth promotion, preventive, metaphylactic, and therapeutic purposes [8]. Even though the use of growth promoters is illegal in other countries, Zambia has a known problem with the non-therapeutic use of antibiotics for prophylaxis and growth promotion in food animal production across many industries [9]. Approximately 75% of the 12 million kg of veterinary antibiotics used globally each year are used to treat infectious diseases, while the remainder are used to promote growth or prevent disease [10]. Antibiotics are chemical substances that belong to a class of natural or synthetic antimicrobial agents that are intended either to kill (bacteriocidal) or inhibit the growth (bacteriostatic) of bacteria. According to a report by [9], Zambia imported 11 metric tons (MT) of antibiotics in 2017 for use in growth promotion, prevention, and treatment. The primary classes of imported antibiotics were penicillins, tetracyclines, sulfonamides, and fluoroquinolones.

Additionally, the recent surge in antibiotic usage in animal source food production has resulted in misuse and overuse due to a lack of user awareness of the consequences of antibiotic residues [11]. In Africa, including developing countries such as Zambia, there has been a high disease burden, inadequate adherence to antibiotic withdrawal periods, and limited veterinary supervision. There is also limited surveillance protocol for antibiotic residues, awareness, inadequate regulations, and knowledge, attitude, and practices (KAP) leading to deposition of antibiotics in animal source food such as milk, eggs, poultry, meat, and fish, contributing to AMR [12,13]. The indiscriminate use of antibiotics constitutes a health hazard for the Zambian population due to non-compliance with the antibiotic withdrawal period [14]. Another major contributing factor to the elevated risk of antibiotic residues in milk is the lack of an efficient food residue monitoring system and insufficient detection capabilities in the majority of developing African nations [15].

The misuse and overuse of antimicrobials such as antibiotics in animal source food production is widely recognized as a key driver of AMR in humans who ultimately consume the animals and animal products. As reported in [9], the issue of uncontrolled use of inadequate dosages of antibiotics for non-therapeutic objectives, such as growth promotion and the prevention of mass disease (prophylaxis), has not been unique to Zambia, a developing country. This is typically more prevalent among smallholder producers who lack antibiotic withdrawal compliance because their ability to survive in business critically hinges on their ability to maximize output and minimize losses.

Numerous reports of antibiotic residues in animal-derived food have surfaced both globally and in Africa as a result of improper antibiotic use with regard to adhering to the antibiotic withdrawal period. These residues are frequently found above the established maximum residue levels (MRLs) in many African nations, which is linked to the emergence of AMR [16]. Antibiotic residues in animal-derived foods are associated with cancer, allergic responses, bone marrow damage, and intestinal microbiota destruction [8,9]. AMR foodborne pathogens were found to be 11% prevalent in sub-Saharan African nations [17]. In Zambia, the burden of AMR foodborne pathogens has significantly increased over a period of five years (2019–2023) when compared with other parts of the world. This increase is explained by the bacteria’s capacity to create defense mechanisms and proliferate readily among people, animals, and the environment. This issue has been made worse due to insufficient management by stakeholders, poor surveillance, and public ignorance, necessitating the health sector and other stakeholders to establish policies [17]. According to a report by [9], a number of studies carried out in Zambia have shown that microorganisms isolated from animal source foods have developed resistance (AMR). Antibiotic residues in milk have also had a detrimental impact on the dairy industry, which is gradually failing owing to economic losses caused by interfering with bacterial cultures required for the production of cheese and yogurt [11,12].

The Global Action Plan (GAP), which was established by the World Health Assembly in 2015 in response to an alarm about the AMR epidemic, intends to combat AMR by enhancing the body of information and evidence through research and surveillance [18]. In 2017, Zambia developed a “One health” collective strategy drawn from the WHO GAP to address the AMR in humans, animals, and the environment [19]. To further address AMR in a coordinated way across many sectors and levels, Zambia also developed a 10-year National Action Plan (NAP) as a framework [9]. The main focus of this NAP is on animal health stewardship initiatives, antimicrobial availability regulation, and optimal antimicrobial usage.

Despite the existing policies and strategies, Zambia still has limited information for local dairy producers regarding antibiotic use and residues, as well as poor regulation, monitoring, and surveillance protocols and systems regarding antibiotic residues [6]. The nature and consequences of antimicrobial use (AMU) in animal production systems in Zambia are still ill-defined [20]. The information regarding the usage of antibiotics and their residues in milk production in the Mbala and Kasama districts is non-existent. These two regions of Zambia’s Northern Province are primarily small-scale milk-producing areas with probably a tendency to maximize milk output and minimize losses by overusing antibiotics. Smallholder dairy producers in both regions do not supply milk to commercial off-takers, who typically require compliance with MRLs for antibiotic residues in milk. This creates a significant risk that farmers may not adhere to withdrawal periods for antibiotic residues in the milk they produce. Moreover, only one study was reported in some parts of Lusaka, Zambia, which evaluated antibiotic residues in bulk raw cow milk from smallholder dairy producers [21]. This circumstance clearly suggests that there is a deficiency in surveillance data regarding antibiotic residues in milk that is produced in Zambia.

The findings from this research present essential evidence for policy and decision-making to address AMR associated with antibiotic residues in milk. Therefore, this study was conducted as part of the core strategies to address AMR through surveillance of antibiotic residues in milk. The Charm II system test is a fast immunoreceptor assay that is intended for use by dairy, veterinary, laboratory, field, and regulatory workers. It identifies several antibiotic class residues at EU/MRL in raw commingled and pasteurized cow milk [22]. This study focused on detecting the presence of five (5) antibiotic class residues, namely: beta-lactams, tetracyclines, sulphonamides, aminoglycosides, and macrolides. The purpose of this study was to assess antibiotic residues in raw cow milk samples from smallholder dairy farms in Mbala and Kasama, Zambia, with a view to ascertaining compliance with established MRLs and to compare prevalence between the two districts.

## 2. Results

### 2.1. Establishment of Critical Control Points for Antibiotic Residue Detection

The critical control point for each class of antibiotic residue was calculated using the Charm II assay protocols, which necessitated the determination of a positive and negative sample (Table 1) [22,23,24,25,26]. Samples having counts per minute (cpm) above the set critical control point were considered negative samples (meaning antibiotic residues were below EU/MRLs), while those having counts per minute (cpm) below the critical control point were considered positive samples (meaning antibiotic residues were above EU/MRLs) in accordance with protocols [22,23,24,25,26]. In order to validate the results, each class of antibiotic residue was run with either a positive or a negative (zero) control standard. The analysis of sulphonamides, tetracyclines, aminoglycosides, and beta-lactams utilized a negative or zero control standard, while macrolides utilized a positive control standard (Table 1).

### 2.2. Occurrence of Antibiotic Class Residues in Raw Cow Milk

The prevalence of five (5) different classes of antibiotic residues in milk samples included 68.8% sulfonamides, 58.1% macrolides, 12.9% tetracyclines, 9.7% beta-lactams, and 2.2% aminoglycosides, as illustrated in Table 2. Approximately 91.4% (*n* = 85) of the 93 milk samples tested had antibiotic residue classes, with mean positive samples being 0.91 ± 0.28. At least one class of antibiotic residues was present in 82.1% of milk samples from Kasama and 98.1% of milk samples from Mbala (*p* = 0.006). Of the five classes, only sulphonamides showed a significant difference between both districts (*p* < 0.001) (Table 2).

### 2.3. Proportion of Milk Samples with Multiple Classes of Antibiotic Residues

Overall, the proportion of milk samples with multiple classes of antibiotic residues was 47.3% (*n* = 44) in both regions. The proportion of milk samples with multiple classes of antibiotic residues was 37.5% (12 out of 32 positive samples) in Kasama and 60.4% (32 out of 53 positive samples) in Mbala district (Table 3). Among milk samples from Kasama with multiple residues, a combination of macrolides and sulphonamides was the highest with 58.3%; followed by tetracyclines and sulphonamides 16.7%; tetracycline, beta-lactams, and sulphonamides 8.3%; tetracycline, macrolides, and sulphonamides 8.3%; and tetracycline, macrolides, beta-lactams, and sulphonamides 8.3%. Among samples from Mbala with multiple residues, a combination of macrolides and sulphonamides was the highest with a 75%; followed by tetracycline and sulphonamides 6.3%; macrolides and beta-lactams 3.1%; macrolides, beta-lactams, and sulphonamides 3.1%; tetracycline, macrolides, beta-lactams, and sulphonamides 3.1%; tetracycline, macrolides, sulphonamides, and aminoglycosides 3.1%; beta-lactams and sulphonamides 3.1%; and beta-lactams, sulphonamides, and aminoglycosides 3.1% (Table 3).

### 2.4. Association Between District and Occurrence of Antibiotic Residues in Milk Samples

This study showed an OR = 0.086 and a significant association (*p* = 0.025) between a district and the occurrence of antibiotic residue classes in milk samples collected from both the Mbala and Kasama districts (Table 4).

## 3. Discussion

To the best of the author’s knowledge, this study presents the first-ever report of antibiotic class residues in raw cow milk sampled from individual lactating cows among smallholder dairy farms in Zambia. This study focused on milk samples collected from individual animals because bulking milk can reduce the levels of antibiotic residues to undetectable levels. Checking for antibiotic residues at the individual cow level helps to ensure that bulk milk collected is safe for human consumption and free of inhibitors at the end of the withdrawal period [27,28,29]. This research successfully identified the critical control points for all five classes of antibiotics, including tetracyclines, sulphonamides, macrolides, beta-lactams, and aminoglycosides, permitting the detection of their residues as either positive or negative presence in relation to EU/MRLs (Table 1). The majority of milk samples tested positive for at least one class of antibiotic residues at or above EU/MRLs in both the Mbala and Kasama districts. Overall, sulphonamides and macrolides were the most prevalent classes of antibiotic residues in the majority of milk samples, a clear indication of their frequent usage in dairy production during the period of sample collection in both regions. Other detected classes included tetracyclines, followed by beta-lactams, and lastly aminoglycosides.

Notably, milk samples from Mbala showed a higher occurrence of antibiotic class residues compared with Kasama (Table 2). This study also reported that Mbala was 11.6 times more likely to have antibiotic residues in milk than Kasama (Table 4). This disparity suggests that, in terms of non-compliance with the antibiotic withdrawal time, Mbala had a higher rate of antibiotic misuse among smallholder dairy farmers than Kasama. Kasama district is a cosmopolitan town with more established agro-vet shops or markets than Mbala, a situation that allows farmers in Kasama to somewhat formally access antibiotics than those in the Mbala district. The study also showed that the occurrence of antibiotic residues in milk was significantly associated with a district (Table 4). This suggested that both districts had smallholder dairy farmers with some similar activities, which led to the deposition of antibiotic residues in milk, despite them showing different proportions of positive milk samples. This study reported the proportion of samples with multiple classes of antibiotic residues in both districts (Table 3). According to this finding, the majority of smallholder dairy farmers in both districts employed a combination of antibiotics or multiple antibiotics within a single treatment course. The most often utilized combination in their dairy production was sulphonamides and macrolides.

Furthermore, the Mbala district reported a higher proportion of samples with multiple classes of antibiotic residues than Kasama. This implies that farmers in Mbala tended to combine drugs during usage more than those in Kasama. Despite the difference between the two districts, antibiotics were utilized in combination in order to possibly achieve better outcomes, a situation that constitutes a public health threat among consumers. Of the five classes, only sulphonamides showed a significant difference between districts. This finding indicates that sulphonamide-based drugs were the most commonly used antibiotics among smallholder dairy producers in the Mbala district compared with the Kasama district, possibly because of easier access and cheaper costs in Mbala than in Kasama. This suggests that sulphonamides were preferred as the drug of choice amongst smallholder dairy farmers in both Mbala and Kasama despite the differences. The prevalence of antibiotic residues in milk samples from both districts was concerning and indicates a possible driver of AMR.

The Charm II system was used to identify milk samples with antibiotic residues at or above EU/MRLs by setting critical control points for each of the five classes of antibiotic residues. The positive milk samples suggested that the antibiotic withdrawal period was not observed, resulting in antibiotic residual deposition at or above EU/MRLs. This poses a threat associated with the emergence of AMR, which is a significant public health threat among consumers. Animal source foods such as milk with antibiotic residues above MRLs are considered not safe for human and animal consumption. Milk samples that tested positive suggested that they had antibiotic residues at or above EU/MRLs, while those that tested negative indicated that antibiotic residues were either detectable but below MRLs or entirely absent. The critical control point was a crucial instrument that indicated adherence to the antibiotic withdrawal period among dairy producers.

Following the detection of antibiotic class residues in milk, the results were compared with those reported by other researchers. The prevalence of antibiotic residues in this study was higher than in a similar study in Zambia [21], which indicated that 30.1% of raw bulk milk samples were positive for antibiotic residues. The results of this study point to a rise in antibiotic overuse among Zambia’s smallholder dairy farmers, who are neglecting to observe the recommended antibiotic withdrawal period because of a lack of awareness, a lack of veterinary supervision during antibiotic use, and inadequate regulatory processes for acquiring antibiotics. A study conducted in Brazil [30] had a prevalence of 0.5% above MRLs, an indication of a low rate of violation of the established regulations for antibiotic use, which was lower than this study’s findings (Table 2). In contrast to Brazil, which has stringent laws governing the use and access of antibiotics, this suggests that dairy farmers in Zambia’s Mbala and Kasama regions are not adhering to the regulations since veterinary medications and services are not adequately regulated. Due to the high veterinary-to-farmer ratio, smallholder dairy producers and other livestock farmers do not receive adequate supervision or service when it comes to antibiotic drugs.

Sulphonamide-based drugs are among the commonly used antibiotics by cattle farmers in Zambia, which agrees with this study’s findings [6]. This study indicated that sulphonamides were the most prevalent in milk samples from both districts, indicating their common use, which strongly suggested their misuse. This could indicate that smallholder dairy farmers commonly use sulphonamides because they are less expensive, and that they are simpler to obtain because of lax regulatory oversight in these districts. A study in Kenya indicated a 2% prevalence of sulphonamides in milk samples above MRLs [31], which contradicted this study’s findings. This finding suggests that smallholder dairy producers in our study used more sulphonamides in disease management than those reported in Kenya [31], who used more beta-lactams and tetracyclines, which are commonly used in disease management for mastitis [32]. The difference in the type of antibiotic residues detected in milk between these two areas indicates differences in the cost of drugs, market access to dairy farmers, and differences in seasonality and livestock diseases, which require different veterinary drugs.

According to this study, macrolides were the second most common antibiotic residue found in milk samples, suggesting that they were used frequently, which led to overuse. The high frequency of macrolides in this study suggests that smallholder dairy farmers in both districts choose to use macrolides as an alternate medication instead of tetracycline, sulphonamides, and penicillins, which are frequently reported to be used by dairy farmers [6]. This study finding is consistent with a study conducted in Guelma, Algeria [33], which identified macrolides as the second most prevalent residues in milk. This suggests that smallholder dairy producers in Guelma, Algeria, and those in this study are increasingly using macrolides as their preferred medication because of lower costs, lax regulatory oversight, and the availability of an alternative drug after prolonged overuse of tetracyclines and beta-lactams. The results of this study were slightly higher than those of a study in Algeria [33], which reported a 65.5% prevalence of penicillin, tetracyclines, macrolides, sulphonamides, and quinolones above MRLs in milk. This suggests that dairy farmers in this study are misusing antibiotics during their usage in their dairy production because of poor regulations and a lack of awareness, than those reported in Algeria.

Although tetracyclines indicated a low prevalence in this study, they are still considered commonly used antibiotics in Zambia among livestock farmers for therapeutic, prophylaxis, and growth promotion [6,29]. This implies a reduction in the use of the tetracycline-based drugs among smallholder dairy producers during this season of sample collection (cold season). The shift to other drugs such as macrolides and sulphonamides suggests that tetracyclines were not commonly used because of long-term use over the recent past, which has made them resistant to most bacteria. The 33% tetracycline prevalence reported in Kenya [31] suggests that Kenyan dairy producers used more tetracyclines than the farmers in this study, which indicates some variations in disease prevalence coupled with medication usage and costs.

In this study, beta-lactams were detected in a small proportion of milk samples despite being commonly used among livestock producers in Zambia [6]. This finding suggests a possible reduction in usage during the time frame (cold season) of milk sample collection from both the Mbala and Kasama districts. Given the widespread use of beta-lactams in recent years, this suggests that farmers favored using alternative medications such as sulphonamides and macrolides. Additionally, this implies that beta-lactams are now more expensive and are therefore less frequently utilized by these smallholder dairy farmers. A study in Kenya [31] indicated that beta-lactams had a prevalence of 10.5% which was similar to this study. This shows that dairy producers in both studies used beta-lactams to treat diseases in similar ways. This implies that after prolonged use, beta-lactams are currently used less frequently than other drugs in both studies.

The least prevalent kind of antibiotic residue found in milk samples was found to be aminoglycosides. This finding indicated its uncommon use among smallholder dairy producers, especially those in Kasama. There were a few milk samples in Mbala that had positive aminoglycoside residue tests, but none in the Kasama region. According to this study, dairy farmers in Mbala also used aminoglycosides as an alternate drug in addition to sulphonamides, macrolides, tetracyclines, and beta-lactams [6], whilst those in Kasama infrequently used aminoglycosides. This suggests that there are differences between the two regions’ dairy practices regarding the use of this medication.

The overall prevalence of antibiotic residues in this study was close to 83% in a study conducted by [34]. The prevalence of antibiotic residues in both studies was high and indicates an indiscriminate use of antibiotic drugs and non-adherence to the antibiotic withdrawal period. This can be due to low awareness and knowledge, negative attitudes, and inappropriate practices regarding antibiotic use and residues in milk, poor regulatory controls, and poor market frameworks for veterinary drugs. This situation poses a significant public health threat to consumers.

Therefore, this study provides a scientific basis that necessitates an urgent intervention strategy through a practicable regulatory framework to strengthen monitoring and surveillance of antibiotic residues and AMR in milk and other animal source foods to assure food safety and public health. Implement routine screening for antibiotic residues in milk using cost-effective and user-friendly tests like microbial inhibition tests. This study was limited to only qualitative analysis of antibiotic residues in milk from Mbala and Kasama, Zambia. This study was also limited to a single sampling season, and no farm-level antibiotic usage data were obtained. This study focused only on the assessment of antibiotic residues in milk and did not include other types of animal source foods. Therefore, additional studies should assess the quantities of antibiotic residues, other antimicrobials, and AMR across different animal source foods in other parts of the country to provide a better national and regional picture. These additional studies should be conducted across different seasons. There is also a need to assess the levels of awareness, knowledge, attitude, and practices on antibiotic use and residues at the farm-level among smallholder dairy producers within the study area and other parts of the country.

## 4. Materials and Methods

### 4.1. Ethical Consideration

Prior to data collection, ethical clearance was sought from the Directorate of Veterinary Services, Ministry of Fisheries and Livestock, Mulungushi House, Lusaka, Zambia (Ref No. DVS101/16/2/2025). The farmer’s informed consent statement was obtained verbally.

### 4.2. Study Design and Areas

**Figure 1:**
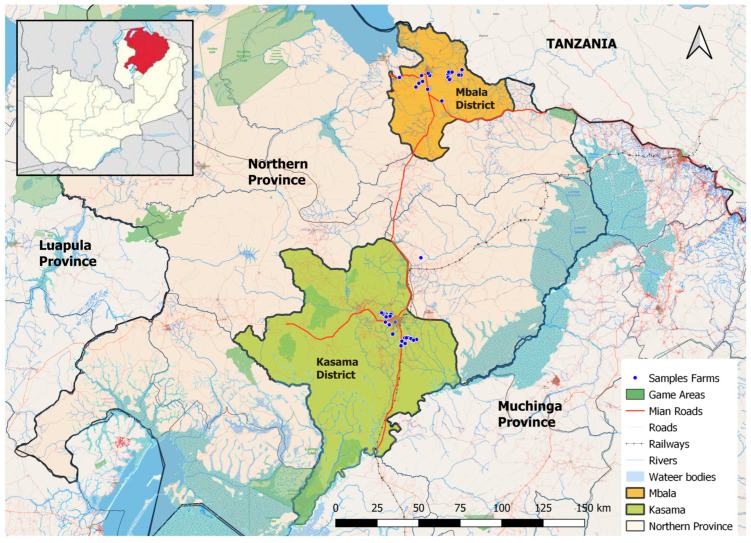
Map showing study sites in Zambia. The map was generated in QGIS Version 3.38.0. The red-coloured region in the above Figure 1 represents the Northern province of Zambia.

A cross-sectional study was conducted, in which 93 milk samples were collected randomly from 93 lactating cows on 56 selected smallholder dairy farms in the Mbala and Kasama districts of Northern Province, Zambia (Figure 1) from May 2025 to June 2025. Milk samples were collected from smallholder dairy farms using a simple random sampling technique. There were 41.9% (*n* = 39) milk samples from Kasama among 36 selected farms and 58.1% (*n* = 54) milk samples from Mbala among 20 selected farms. This study encompassed all 56 smallholder dairy farms that were actively engaged in milk production during the study period. At least one milk sample was randomly collected per smallholder dairy farm. Kasama and Mbala districts were purposively selected because they had the highest number of daily cattle actively producing milk at the time of this study. Additionally, the province lacked commercial off-takers, who typically require compliance with MRLs for antimicrobial residues in milk. Therefore, this situation created a high likelihood of antibiotic misuse and overuse.

### 4.3. Study Population and Sample Size

A sampling framework was obtained from the district livestock stock registers in both the Kasama and Mbala districts. A total of 103 lactating cows constituted the study population (Kasama had *n* = 43 and Mbala had *n* = 60).

The sample size for milk samples per district was estimated with EpiTools epidemiological calculators (https://epitools.ausvet.com.au/?page=home) using the standard sample size formula called Cochran’s formula for estimating a single proportion from simple random sampling [35].

Sample size; n = (Z^2^ × P × (1 − P))/e^2^

The estimated prevalence (P) of antibiotic residues in milk (used 50% since no previous data existed, which yielded a maximum sample size) [35]. Although the calculated sample size was 91, this study managed to collect 93 samples.

### 4.4. Milk Sample Collection

The animal was first restrained in a crush pen, and then the udder was washed with clean water. A 100 mL milk sample was then aseptically collected into a sterile plain bottle [13,33]. The bottles were properly labeled with an allocated animal ID, date, and then placed in the cooler box containing ice packs at 2 °C to 4 °C and transferred to the freezer at the district laboratory, where they were stored at −18 °C to −20 °C within 4 h after collection [13]. Samples were then transported at −18 °C to −20 °C to the Central Veterinary Research Institute (CVRI), Lusaka, Zambia, within five (5) days from the day of sampling to analysis. The samples at CVRI were kept in a freezer at −20 °C for 24 h before the start of analysis [36] using the Charm II system [22,23,24,25,26]. No preservatives were added to the milk to prevent bacterial growth.

### 4.5. Milk Sample Analysis with Charm II System

The protocols obtained from Charm Sciences Inc., Lawrence, MA, USA, were used to guide the screening of five (5) different classes of antibiotic residues in milk matrix, namely: beta-lactams, tetracyclines, sulphonamides, aminoglycosides, and macrolides [22,23,24,25,26]. The Charm II system test for tetracycline detection had a sensitivity of 90%, whereas the corresponding sensitivities for macrolides, beta-lactams, sulphonamides, and aminoglycosides were 90%, 90%, 99%, and 90% respectively [22,23,24,25,26]. Before each analysis, a specified number of frozen milk samples were placed in a rack at room temperature for approximately 2–3 h to allow thawing and then gently swirled up and down 20 times for 20 s, allowing mixing.

#### 4.5.1. Principles of the Charm II System

The Charm II test used a binding reagent with specific receptor sites that bind antibiotic drugs. The binder was added to a sample along with an exempt amount of [14C] or [3H] labeled antibiotic tracer. Any antibiotic in the sample competed for the binding sites with this tracer. The amount of tracer that binds to the receptor sites was measured and compared with a previously determined critical control point. The critical control point was the cutoff point between a negative and a positive sample. The greater the amount of tracer measured, the lower the antibiotic concentration in the sample. The smaller the amount of tracer measured, the greater the antibiotic concentration in the sample [22,23,24,25,26].

#### 4.5.2. Validation

The methods for equipment cleanup, setup, and detection of all five (5) classes of antibiotic residues from milk samples were optimized and validated in compliance with protocols from Charm Sciences Inc., MA, USA [22,23,24,25,26]. The set critical control points for sulphonamides, tetracyclines, aminoglycosides, and beta-lactams were validated by comparing with the counts per minute (cpm) for one (1) zero control standard, while the critical control point of macrolides was validated by comparing with the counts per minute (cpm) for one (1) positive control standard (Table 1). In this study, the protocols were slightly modified during validation of critical control points by comparing with only one (1) zero or positive control standard instead of three (3) [22,23,24,25,26]. This was due to the limited availability of tracer and binder tablets.

#### 4.5.3. Quality Control Samples and Calibration

Quality control samples containing antibiotic-free milk (negative or zero control standard) and milk containing antibiotics; 4.00 ± 0.15 ppb penicillin G or 10.0 ± 0.4 ppb sulfamethazine or 30 ppb oxytetracycline or 30 ppb gentamicin or 50 ppb erythromycin (positive control standards) were supplied with the kit and manufactured from USP reference standard, Charm Sciences Inc., 659 Andover Street, Lawrence MA 01843-1032 USA [22,23,24,25,26]. These control standards were stored at −20 °C and were utilized during positive and negative control preparation and the validation process. The Charm II system was calibrated by entering the determined critical control point into the Charm II control panel prior to the detection of each class of antibiotic residue (inter-assay).

#### 4.5.4. Interpretation of Charm II Results

The Charm II system was used to identify milk samples with antibiotic residues at or above EU/MRLs by setting critical control points for each of the five classes of antibiotic residues according to Charm II system protocols [22,23,24,25,26]. Samples with a cpm value above the critical control point were considered negative, while those with a cpm value at or below the critical control point were considered positive. However, milk samples that tested positive indicated that they had antibiotic residues at or above EU/MRLs, while those that tested negative indicated that antibiotic residues were either detectable but below EU/MRLs or entirely absent.

#### 4.5.5. Charm^®^ II Beta-Lactam Test [25]

##### Zero Control Standard Preparation

A zero control standard milk powder was included in the kit, certified organic whole milk powder, to be antimicrobial drug-free. It was reconstituted with 100 mL of 40 °C water as indicated on the label and was shaken well (until all clumps were broken up). Then, it was cooled by refrigeration before use, and the remaining dry standard was refrigerated. The reconstituted standard was held refrigerated for up to 48–72 h [25]. This procedure for negative control standard preparation was applied to all the other four (4) antibiotic classes [22,23,24,26].

##### Positive Control Standard Preparation

A multi-antimicrobial standard (containing 4.00 ± 0.15 ppb penicillin G when reconstituted) supplied with the kit, manufactured from USP reference standards, MA, USA, was used as a positive standard [25]. This standard was reconstituted with 50 mL negative control—zero control standard. The mixture was shaken well and allowed to stand refrigerated for 15 min, and mixed before use. The dry standard was stored and refrigerated, and the reconstituted standard was held refrigerated for up to 48 h [25].

##### Beta-Lactam Test Procedure for Control and Sample

A green tablet was added to the empty test tube, and 300 μL of water was added and mixed using a vortex (model MX-5) for a minimum of 10 s to completely break up the tablet. Then, 5.0 mL of positive control was added and immediately mixed using a vortex (model MX-5) for 10 s. This mixture was incubated at 65 °C for 2 min, and a yellow tablet was added, which contained less than 0.2 kilobecquerels (kBq) of [14C]-penicillin G, and was immediately mixed by swirling milk up and down 10 times for 10 s. This mixture was incubated (Inctronic 2) at 65 °C for 2 min and centrifuged (Rotofix 32A) for 3 min at 3400 rpm [25]. Then, it was immediately removed from the centrifuge, and the milk was decanted. The fat ring was removed and wiped dry with a cotton swab without disturbing the pellet. Then, 300 μL of water was added and mixed using a vortex (model MX-5) thoroughly to break up the pellet and ensure it is suspended in water before adding scintillation fluid. A volume of 3.0 mL of scintillation fluid was added, and the tube was capped and mixed using a vortex (model MX-5) until the mixture had a uniform cloudy appearance. Then the sample was counted in an analyzer (Charm II 6600 counter version 3.12) for 60 s. The cpm (count per minute) was read on the [14C] channel. If the sample exceeded or was within 50 cpm of the critical control point, it was recounted [25]. The same approach was used in all milk sample analysis.

##### Critical Control Point Setup

Six replicates of the positive control standards were run, and the average was determined. Thereafter, 15% was added to the positive average; this was the critical control point [25]. The critical control point is valid if all six determinations are within 15% of the positive average. Single deviants were retested and re-averaged. If more than one deviant value was obtained, the critical control point determination was performed again [25]. Samples were run with only one zero control standard for validation.

#### 4.5.6. Charm^®^ II Sulphonamides Test [22]

##### Positive Control Standard Preparation

A 100 mL 10.0 ± 0.4 ppb sulfamethazine standard was supplied with the kit and manufactured from a USP reference standard, MA, USA. The sulfamethazine standard was reconstituted with 100 mL of negative control (zero control standard), shaken well, and allowed to stand while refrigerated for 15 min before use. The reconstituted sulfamethazine standard was stored at 0 to 4.4 °C for up to 48 h [22].

##### Sulphonamides Test Procedure for Control and Sample

A white tablet was added to an empty test tube, and 300 μL of water was added. The solution was mixed for a minimum of 10 s using a vortex (model MX-5) until the tablet was dissolved. A volume of 5.0 mL of positive control standard was added, and then a pink tablet was added (contains 3.0 kilobecquerels (kBq) of [3H]-sulfamethazine) and immediately mixed with a vortex mixer (model MX-5) or by swirling the milk up and down 15 times for 15 s. This mixture was incubated (Inctronic 2) at 85 °C for 3 min [22], centrifuged (Rotofix 32A) for 3 min at 3400 rpm, and immediately removed from the centrifuge, and the milk was poured off. The fat ring was removed and wiped dry with swabs without disturbing the pellet. A volume of 300 μL of water was added and mixed thoroughly using a vortex mixer (model MX-5) to break up the pellet to ensure it was suspended in water before adding scintillation fluid. A volume of 3.0 mL of scintillation fluid was added, and the tube was capped and mixed using a vortex mixer until the mixture had a uniform cloudy appearance. Then, the sample was counted in an analyzer (Charm II 6600 counter version 3.12) for 60 s. The cpm (count per minute) reads were obtained on the [3H] channel [22]. The same approach was used in all sample analysis.

##### Critical Control Point Setup

Six replicates of the positive control standard were run, and the six cpm results were averaged. If any of the six determinations deviated more than 15% from the average, then it was replaced in the determination and averaged again. A total of 24% was added to the average to calculate the critical control point [22]. Only one zero control standard was run for validation.

#### 4.5.7. Charm^®^ II Tetracycline Test [23]

##### Positive Control Standard

The multi-antimicrobial standard (containing 30 ppb oxytetracycline when reconstituted) supplied with the kit was manufactured from USP reference standards, MA, USA. The multi-antimicrobial standard was reconstituted with 100 mL negative control—zero control standard [23], or raw or pasteurized tetracycline drug-free milk, and was shaken well to mix and allowed to stand refrigerated for 15 min before use. The material was stored dry, while the positive control standard was refrigerated. After reconstitution, the positive control standard was refrigerated for up to 48 h [23].

##### Tetracycline Test Procedure for Control and Sample

All samples were mixed well before testing, and a white tablet was added to the empty test tube. A 300 µL volume of water was added and mixed using a vortex mixer (model MX-5) for 10 s to completely break up the tablet. Then, 5.0 mL of positive control was added, followed by an orange tablet (1.85 kilobecquerels (kBq) of [3H]-tetracycline). This mixture was immediately mixed using a vortex mixer (model MX-5) or by swirling milk up and down 15 times for 20 s [23]. Then, it was incubated (Inctronic 2) at 35 °C for 3 min, centrifuged (Rotofix 32A) for 5 min at 3400 rpm, and immediately removed from the centrifuge. The milk was poured off, and removed the fat ring and wiped dry with cotton swabs while not disturbing pellet. A volume of 300 µL of water was added and thoroughly mixed using a vortex mixer (model MX-5) to break up the pellet. To one tube at a time, 3.0 mL of scintillation fluid was added, and the tube was capped and inverted (or shaken), and mixed using a vortex mixer (model MX-5) until the mixture had a uniform cloudy appearance. Then, the sample was counted in an analyzer (Charm II 6600 counter version 3.12) for 60 s, and the cpm (count per minute) read was obtained on the [3H] channel. If the count was greater than or within 50 cpm of the critical control point, the sample was recounted [23]. The same approach was used in all sample analysis.

##### Critical Control Point Setup

Six replicates of the positive control standard were run, and the six cpm results were averaged. If any of the six determinations deviated more than 23% from the average, then that sample determination was replaced and the runs re-averaged. A total of 23% was added to the average to calculate the critical control point [23]. Only one zero control standard was run for validation.

#### 4.5.8. Charm^®^ II Aminoglycosides Test [26]

##### Positive Control Standard

The multi-antimicrobial standard (containing 30 ppb gentamicin when reconstituted) supplied with the kit was manufactured from USP reference standards, MA, USA. The multi-antimicrobial standard was reconstituted with 100 mL negative control—zero control standard or raw or pasteurized aminoglycoside-free milk comparable [26]. The mixture was shaken well and allowed to stand refrigerated for 15 min. It was mixed before use, and the dry multi-antimicrobial standard was stored dry and refrigerated. The reconstituted positive control standard was held refrigerated for up to 48 h [26].

##### Aminoglycoside Test Procedure for Control and Sample

A test tube was filled to ¾ full and thoroughly mixed with the positive control. Then, it was centrifuged (Rotofix 32A) for 3 min at 3400 rpm, and the sample was cooled to 4 °C. A white tablet was added to the empty test tube. A volume of 300 µL of water was added and mixed using a vortex mixer (model MX-5) for 10 s to completely break up the tablet. A volume of 5.0 mL of centrifuged positive control was added from below the fat layer. Thereafter, a yellow tablet was added (contains 1.9 kilobecquerels (kBq) of [3H]-gentamicin) and immediately mixed using a vortex mixer or by swirling the milk up and down 15 times for 15 s [26]. The sample was incubated (Inctronic 2) at 35 °C for 3 min and centrifuged (Rotofix 32A) for 3 min at 3400 rpm. Then, it was immediately removed from the centrifuge, and removed the fat ring, and wiped dry with cotton swabs and did not disturb the pellet. A volume of 300 µL of water was added and mixed thoroughly using a vortex mixer to break up the pellet, while the pellet was suspended in water before adding scintillation fluid. A volume of 3.0 mL of scintillation fluid was added, the tube was capped and mixed using a vortex mixer (or shaken) until the mixture had a uniform cloudy appearance. Then, the sample was counted in an analyzer (Charm II 6600 counter version 3.12) for 60 s, and the cpm (count per minute) reads were obtained on the [3H] channel. If the read count was greater than or within 50 cpm of the critical control point, the sample was recounted [26]. The same approach was used in all sample analysis.

##### Critical Control Point Setup

Five replicates of the positive control standard were run, and the five cpm results were averaged. If any of the five determinations deviated more than 20% from the average, it was replaced in that determination and re-averaged. A total of 20% was added to the average to calculate the critical control point [26]. Only one zero control standard was run for validation.

#### 4.5.9. Charm^®^ II Macrolides Test [24]

##### Positive Control Standard

A multi-antimicrobial standard (containing 50 ppb erythromycin when reconstituted) supplied with ETBL-kits was manufactured from USP reference standards, MA, USA. It was reconstituted with 50 mL of negative control—zero control standard or raw or pasteurized macrolide/lincosamide-free milk comparable [24]. The mixer was shaken well and allowed to stand refrigerated for 15 min, and mixed before use. Then, the stored dry positive control standard was refrigerated, while the reconstituted positive control standard was refrigerated for up to 48 h [24].

##### Macrolide Test Procedure for Control and Sample

A white tablet was added to an empty test tube, and then 300 µL of water was added and mixed using a vortex mixer (model MX-5) for 10 s to completely break up the tablet. Thereafter, 5.0 mL of negative or zero control was added and immediately mixed using a vortex mixer or by swirling milk up and down 10 times for 10 s and incubated (Inctronic 2) at 65 °C for 2 min. Then, a green tablet (contains less than 0.3 kilobecquerels (kBq) of [14C]-erythromycin) was added and immediately mixed using a vortex mixer or by swirling milk up and down 10 times for 10 s (tablet addition and mixing of all samples was completed within 40 s) [24], incubated (Inctronic 2) at 65 °C for 2 min and centrifuged (Rotofix 32A) for 3 min at 3400 rpm. The sample was immediately removed from the centrifuge, and the milk was poured off. The fat ring was removed and wiped dry with cotton swabs, taking care not to disturb the pellet. A volume of 300 µL of water was added and mixed thoroughly using a vortex mixer to break up the pellet. A volume of 3.0 mL scintillation fluid was added, and the tube was capped and mixed using a vortex mixer (or shaken) until the mixture had a uniform cloudy appearance. The sample was counted in an analyzer (Charm II 6600 counter version 3.12) for 60 s, and the cpm (count per minute) read obtained on the [14C] channel [24]. The same approach was used in all sample analysis.

##### Critical Control Point Setup

Six replicates of the zero control standard were run, and the six cpm results were averaged. If any of the six determinations deviated more than 15% from the average, then it was replaced and the determination was re-averaged. A total of 35% was subtracted from the average to calculate the critical control point [24]. Only one positive control standard was run for validation.

### 4.6. Data Management and Analysis Methods

Laboratory data were entered on a Microsoft Excel worksheet^®^ 2010 and exported to Stata 14.2 for analysis [37]. This study utilized the Pearson chi-square test to assess statistical significance concerning antibiotic residues between the Mbala and Kasama districts, and binary logistic regression was used to show a relationship between a district and the occurrence of antibiotic residues in milk. In this study, descriptive analysis was employed to summarize the collected data and express it in terms of mean, standard deviation, and percentages (%). During analysis, a *p* = 0.05 (Confidence Interval 95%) was used as the cutoff point for significance of difference as described in [13].

## 5. Conclusions

In conclusion, this study highlighted the high occurrence of antibiotic residues in milk samples at or above EU/MRLs, with some samples containing multiple antibiotic residues. This outcome suggests a possible driver of AMR within Zambia. According to this study, the proportion of milk samples that tested positive for antibiotic residues varied significantly between the two districts, with Mbala having a larger proportion than Kasama. The high presence of antibiotic residues in milk samples strongly suggests failure to follow the antibiotic withdrawal period, indiscriminate use and management of antibiotics, and poor regulatory practices among smallholder dairy producers in both districts.

Therefore, there is a need for establishing a strict regulatory policy framework for antimicrobial drug use among livestock producers, routine monitoring, and surveillance of antimicrobial drug residues and AMR in animal food matrices. Additionally, it is essential to educate livestock farmers about antimicrobial stewardship, public health risks connected to residues exceeding MRLs, and the importance of adhering to antibiotic withdrawal periods.

## Figures and Tables

**Table 1:** Establishment of critical control points (CCP) for five classes of antibiotic residues.

Class of Antibiotic	No. of Standards Used	Type of Control Used	Standard Counts per Minute (cpm)	cpm Selected for Averaging	Average cpm	Inclusion or Exclusion (%) to Average cpm	Critical Control Point	Zero Control (cpm)	Positive Control (cpm)
Tetracycline	6	Positive	918; 1019; 965; 1028; 907; 990	918; 1019; 965; 1028; 907; 990	971	25% addition	1194	2065	
Macrolides	6	Negative	1200; 1253; 1257; 1321; 2121; 1245	1200; 1253; 1257; 1321; 1245	1255	35% subtraction	815		724
Beta-lactams	6	Positive	418; 429; 456; 279; 193; 151	418; 429; 456	434	15% addition	499	1501	
Sulphonamides	6	Positive	490; 585; 545; 547; 603; 555	585; 545; 547; 555	558	24% addition	692	1150	
Aminoglycosides	5	Positive	521; 514; 583; 609; 589	521; 514; 583; 609; 589	563	20% addition	676	1386	

**Table 2:** Occurrence of antibiotic class residues % (*n*) in milk samples collected from the Mbala and Kasama districts.

District	% (*n*) Overall Tested Samples	% (*n*) Overall Positive Samples	% (*n*) Positives Antibiotic Classes				
			Tetracycline	Macrolides	Beta-Lactams	Sulphonamides	Aminoglycosides
Mbala	58.1 (54)	98.1 (53)	7.4 (4)	59.3 (32)	11.1 (6)	87.0 (47)	3.7 (2)
Kasama	41.9 (39)	82.0 (32)	20.5 (8)	56.4 (22)	7.7 (3)	43.6 (17)	0 (0)
Total	100 (93)	91.4 (85)	12.9 (12)	58.1 (54)	9.7 (9)	68.8 (64)	2.2 (2)
*p*-Value		0.006	0.063	0.784	0.122	0.000	0.224
Pearson chi2 (χ^2^)		7.4630	3.4608	0.0755	2.3968	19.9200	1.4762
EU/MRLs			100 µg/kg	40–200 µg/kg	4 µg/kg	100 µg/kg	100 µg/kg

Significant *p*-Value < 0.05; X^2^ = Pearson chi-square test.

**Table 3:** Proportion of milk samples with multiple classes of antibiotic residues.

Kasama District		Mbala District	
Classes of Antibiotic Residues	No. of Samples with Multiple Residues	Classes of Antibiotic Residues	No. of Samples with Multiple Residues
Macrolides and sulphonamides	7	Macrolides and sulphonamides	24
Tetracyclines and sulphonamides	2	Tetracycline and sulphonamides	2
Tetracycline, beta-lactams, and sulphonamides	1	Macrolides and beta-lactams	1
Tetracycline, macrolides, and sulphonamides	1	Macrolides, beta-lactams, and sulphonamides	1
Tetracycline, macrolides, beta-lactams, and sulphonamides	1	Tetracycline, macrolides, beta-lactams, and sulphonamides	1
		Tetracycline, macrolides, sulphonamides, and aminoglycosides	1
		Beta-lactams and sulphonamides	1
		Beta-lactams, sulphonamides, and aminoglycosides	1

**Table 4:** Association between district and occurrence of antibiotic residues in milk using binary logistic regression.

District	Antibiotic Occurrence in Milk	Odds Ratio	*p*-Value
Mbala and Kasama	85 Positives	0.086	0.025
	8 Negatives		

## Data Availability

The original contributions presented in this study are included in the article. Further inquiries can be directed to the corresponding authors.

## Abbreviations

The following abbreviations are used in this manuscript:

µL	Microliter
ACF	Agriculture Consultative Forum
AMR	Antimicrobial Resistance
AMU	Antimicrobial use
CAC	Codex Alimentarius Commission
CBPP	Contagious Bovine Pleuropneumonia
cpm	Count Per Minute
CVRI	Central Veterinary Research Institute
EC	European commission
EFSA	European Food Safety Authority
et al.	And others
EU	European Union
FAO	Food Agriculture Organization
HLB	Hydrophilic–Lipophilic Balance
HPLC	High Performance Liquid Chromatography
i.e.,	That is
kBq	Kilobequerels
LC	Liquid Chromatography
LFM	Laboratory Fortified Matrix
LOD	Limit of Detection
LOQ	Limit of Quantification
LRB	Laboratory Reagent Blank
mL	Milliliter
MRLs	Maximum Residue Limits
MS	Mass spectrometer
NAP	National Action Plan
NEPAD	New Partnership for Africa’s Development
NGOs	Non-governmental Organizations
rpm	Revolution per minute
SDGs	Sustainable Development Goals
SPE	Solid-Phase Extraction cartridge
TLC	Thin-layer Chromatography
UV	Ultra-Violet
WHO	World Health Organization
